# The Swedish Cervical Screening Cohort

**DOI:** 10.1038/s41597-024-03519-2

**Published:** 2024-06-26

**Authors:** Joakim Dillner, Laila Sara Arroyo Mühr, Sara Nordqvist Kleppe, Jiangrong Wang, Helena Andersson, Miriam Elfström, Roxana Merino, Karin Sundström

**Affiliations:** 1 Center for Cervical Cancer Elimination, Department of CLINTEC, Karolinska Institutet, F46, S-14186 Stockholm, Sweden; 2 Center for Cervical Cancer Elimination, Medical Diagnostics Karolinska, Karolinska University Hospital, F46, S-14186 Stockholm, Sweden; 3 Clinical Pathology and Cancer Diagnostics, Medical Diagnostics Karolinska, Karolinska University Hospital, F46, S-14186 Stockholm, Sweden

**Keywords:** Cervical cancer, Cancer screening, Preventive medicine, Cancer prevention

## Abstract

The Cervical Screening Cohort enrols women screened for human papillomavirus (HPV) and cervical abnormalities within the capital region of Sweden from the organised screening program and the non-organised testing of cervical samples. The cohort started in 2011 and has enrolled more than 670,000 women, contributing with more than 1.2 million biobanked samples. The cohort is systematically updated with individual-level data from the Swedish National Cervical Screening Registry (NKCx). Key variables include birthdate, sampling date, cytological, histopathological and HPV analysis results, and invitation history. Each sampling and subsequent clinical follow-up is sequentially registered, allowing for longitudinal analyses of screening results and associated results of the clinical workup. The cohort is ideal for longitudinal, long-term follow-up studies due to its validated documentation and registry-derived information. From the data, it is possible to penetrate important human health mechanisms. The data are available as open-data and GDPR-compliant. Samples are available after getting the required permissions. Results will help researchers understand factors that increase cancer risk and other diseases.

## Background & Summary

The Swedish cervical screening program’s primary purpose is to prevent cervical cancer by detecting and removing precancerous lesions, which, if left untreated, could develop into invasive cervical cancer. The program started in 1964 and expanded to a national program between 1967 and 1973. Today, the new recommendation states that every resident woman in Sweden will be called for cervical screening every five years between the ages of 23 and 49 years and every seven years between the ages of 50 and 70^1,2^.

In 2011, the central cervical screening laboratory of the Stockholm region (today, Center for Cervical Cancer Elimination (CCCE)) started archiving the residual liquid-based cytology samples at −30 °C^3^. Comprehensive archiving of samples taken using the older sampling method (Pap smears on glass slides) had started in the 1960s. After the switch to liquid-based cytology (LBC) in the routine program, protocols for archiving cell samples were updated. Samples are now stored at −30 °C in a liquid preservative where the cells do not freeze^3^, and the samples have many more uses for research and development than the previous archival glass slides. Therefore, since 2011, the cervical screening cohort has been defined as a cohort of LBC samples. As of 2023, the cohort contains more than 1.2 million samples taken from over 670,000 women. The annual growth of the cohort varies with the extent of cervical screening (paused during the COVID-19 pandemic). It has ranged between 40,000 to 200,000 new samples added yearly according to sampling occasions and indications. Most women have contributed, on average, with two samples between 2011 and 2022. The enrolment process uses written invitations and informed consent, including an opt-out procedure^4^.

Samples from this cohort have been used in studies identifying cervical DNA-methylation signatures that can predict female cancers such as breast cancer^5^, endometrial cancer^6,7^, and cervical cancer^8,9^ using a single cervical sample. Analysing the cohort’s cervical samples has also shown that among the few HPV-vaccinated women developing cervical intraepithelial neoplasia (CIN) lesions, most lesions are typically associated with non-vaccine HPV types^10^. As part of quality assurance of primary HPV testing in an organised cervical screening programme, laboratory audits use samples from the cohort. Cohort samples taken from women before they were diagnosed with histopathological confirmed CIN3 or worse and tested for HPV or having an HPV-negative result were analysed or re-analysed, respectively, for HPV to evaluate the quality of the HPV-test laboratory procedure^11,12^. In addition, HPV testing systems have been validated with samples from this cohort^13^.

**Fig. 1:**
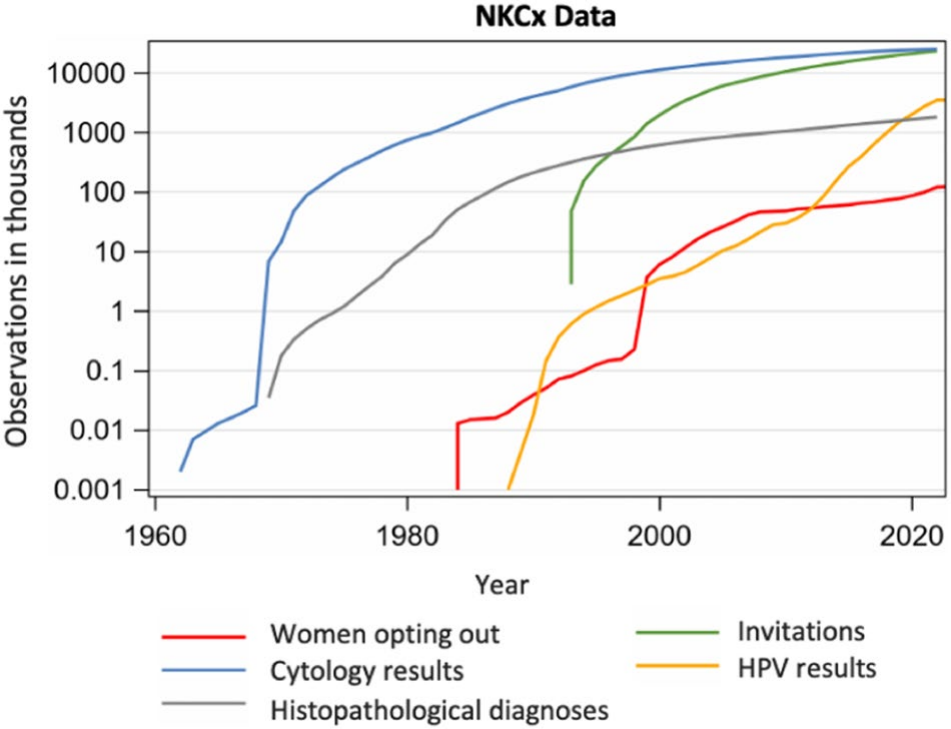
The total number of individually identifiable entries in the Swedish National Cervical Screening Registry NKCx. Historically most entries used to be cytology results, but in recent years most entries are HPV test results. Cytologies, histopathologies and HPV tests are complete since 1995, with partial data registered before that. Individually identifiable invitations and requests to opt out of the screening program are completee since 2005, with partial data registered before that.

The Swedish National Cervical Screening Registry, NKCx, is a formalised part of the cervical screening program’s evidence-based surveillance and quality assurance^14^. NKCx stores data on results from all cervical tests (HPV, cytology, histopathology samples) and data on all issued personal invitations and consents. All units in the country that perform tests or issue invitations submit data annually to NKCx, resulting in 100% national coverage. Key quality indicators (e.g., population invitational coverage, test coverage of screening, diagnostic profiles, and proportion of screen-positive women followed up according to guidelines) and basic statistics are compiled and reported annually to each region’s cervical screening program to improve care delivery^15,16^. Figure 1 shows screening data from 1964 to 2021. The NKCx monitors all invitations (including exact date issued) and screening coverage and participation after invitation each year and by region, which enables comprehensive measurement of non-attendance annually. Regions where non-attendance is higher are in this way alerted to this fact and women who are so-called long-term non-attenders are reminded and re-invited systematically^15,16^. Through linkage to the Swedish Tax Authority and/or Statistics Sweden, the demographical agency of Sweden, correlated data on emigration, immigration and death date are captured such that accurate measurement of person-time for epidemiological studies can be performed.

The Swedish Cervical Screening Cohort can help evaluate exposure risk factors (virus, bacteria, others) in women as measured in cervical exfoliated cells. The cohort contains samples and data from women with a healthy cervix, pre-neoplastic lesions, and cancer. The data are also linkable to a number of other Swedish national health registries for research on disease outcomes occurring among the individuals contributing samples in the biobank, through use of the personal identification number (PIN) that all Swedish citizens including immigrants receive. (Analyses are then carried out under code, i.e. under pseudonymized conditions such that no individual can be identified in the dataset.) These health registries include resources such as the National Cancer Registry, the National Patient Registry and the Medical Birth Registry at the National Board of Health and Welfare as well as the HPV vaccination registries at Public Health Agency of Sweden.

The analysis of specimens and the associated metadata contribute to identifying and characterising factors that may impact the risk of having a disease or predict that the individual will remain healthy. Although the cohort contains samples only from women, the cohort is uniquely suited for research projects aiming to elucidate any disease mechanism which functions similarly in both genders. The female origin of samples thus does not preclude the possibility of generalising findings on, e.g., risk factors of specific genetic factors, also to men. For example, insights gained into how HPV infection causes cervical cancer can also extend to anal cancer, tonsillar cancer and other cancer forms affecting both genders.

Pseudo-anonymised data can furthermore enrich datasets to train and validate machine learning models to classify or predict cervical cancer and all metagenomics analysis pipelines developed by studies on the Cervical Screening Cohort^17,18^ are open source. These pipelines can thus enrich other datasets with evidence based on metagenomics approaches.

The Cervical Screening Cohort (CSC) is part of the proof of concept for the Human Exposome Assessment Platform project (HEAP)^19^, one of the nine projects from the European Human Exposome Network (https://www.humanexposome.eu/).

## Methods

The CSC contains stored cervical cells from almost the entire screening population of women in the capital region of Stockholm, Sweden. This region corresponds to around 20% of the nation. For those women who previously participated in cervical screening outside of Stockholm, the CSC has access to all diagnostic results from such tests through updates from NKCx. There are some other regions such as Örebro and Umeå in the nation, which systematically biobank screening sample residuals. If a woman has previously attended screening in such regions, her sample will have been biobanked and a aliquot will be retrievable upon request from said other regional biobanks as well.

**Fig. 2:**
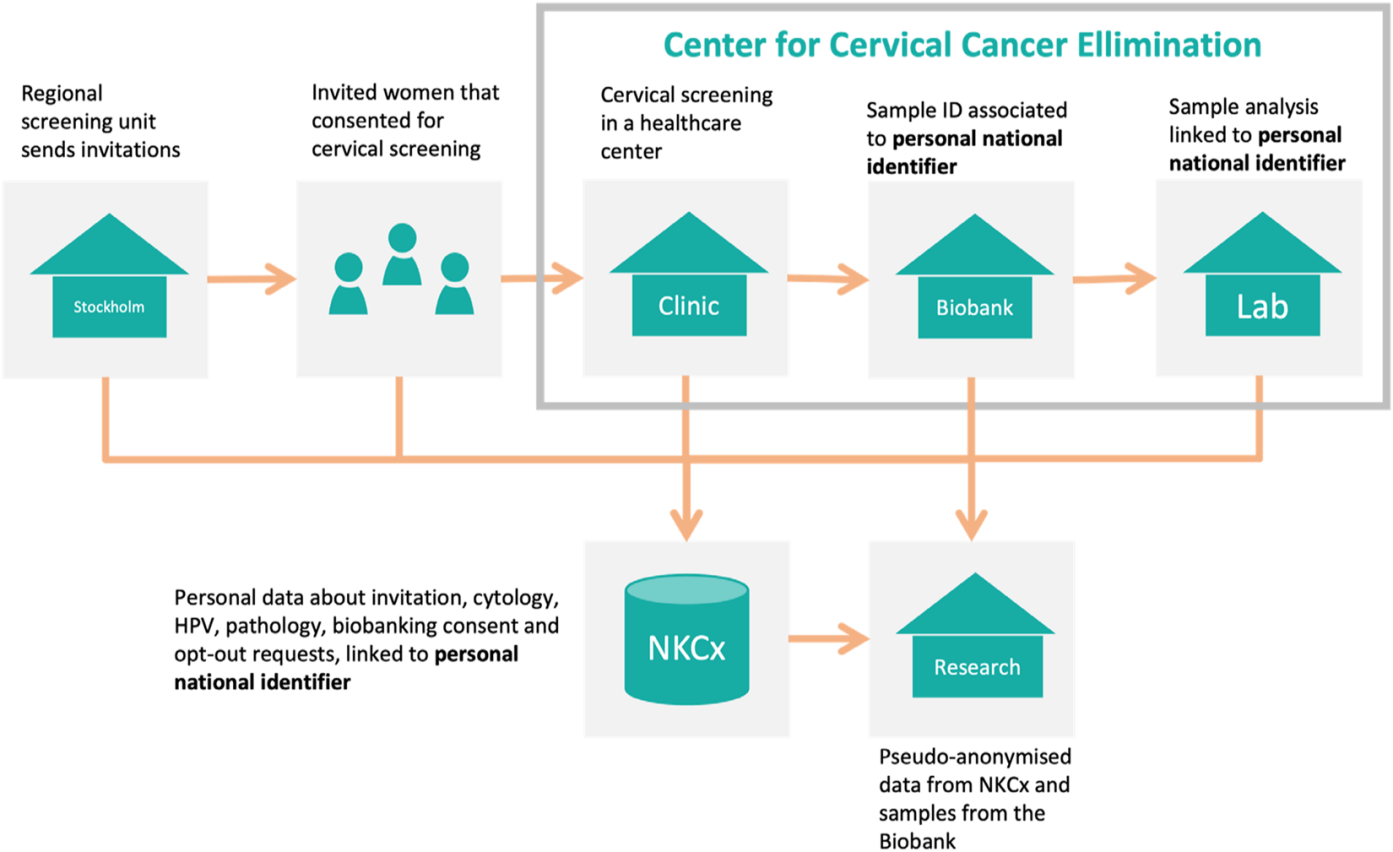
The organisation of the Swedish Cervical Screening Cohort. The Center for Cervical Cancer Elimination collects the cervical biospecimens and adds associated data from the Swedish National Cervical Screening Registry.

The follow-up of participating women allows for identifying whether they developed cervical cancer precursors, cancer, or no cancer. Analysing specimens and metadata makes it possible to identify and characterise factors associated with the presence or absence of disease. The main steps in the screening process are illustrated in Fig. 2.

Main steps of the cervical screening process:

1. The regional screening program issues invitations to women resident in the Stockholm area, the capital region of Sweden, using a risk-stratified screening procedure involving the woman’s age and screening history, including HPV test result status. The invitation contains information about the screening procedure, the time and place for sampling, and how to opt out from storage. It also specifies that the samples and data will be stored and used for quality assurance and research. Very few women opt out to have their sample stored and/or used for research (<20 per year).
2. Women consenting to participate in the screening program follow the instructions and visit the healthcare unit for sampling.
3. The samples are sent to the CCCE for registration, storage, and analysis. Each sample has a unique identifier linked with the woman’s national identification number (PIN). The PIN includes the birthdate of the woman. A Lab Information Management System (LIMS) keeps data about the sampling date, sample storage location, and storage date.
4. All samples are analysed by PCR to detect oncogenic HPV types. The result of the analysis is stored, linked to the sample, and exported to the NKCx. All results from follow-up visits including triage are also stored, in terms of the pathology result. The type of procedure that yielded the pathology results (such as colposcopy and/or endometrial sampling/curettage) is available through medical records if needed, but are not recorded in the NKCx or the cohort.
5. A notification is sent to the screening participants to inform them about the result. If the analysis result is positive for the high-risk HPV types, age and HPV type-specific follow-up protocols are applied according to the national standard of care. Cervical samples and biopsies taken during follow-up are sent to the lab for analysis, and the results are registered in NKCx.
6. The NKCx publishes annual reports regarding all invitation, cytology, HPV, pathology, biobanking consent and opt-out requests in Sweden. The PIN is the unique identifier that links all the data to a woman. The cervical screening cohort participant’s information is updated with NKCx data annually.
7. After being granted access, a researcher can use data from the NKCx registry and samples from the CCCE lab.

Apart from the samples in the screening program, the cohort also contains all samples and data from non-organised cervical tests, for example, clinically indicated tests taken because of symptoms suspicious of cervical cancer. All samples deriving from organized screening are labelled by the sample taker with a certain prefix, in the most recent years examples are Y (previous program with primary cytology), YS (current program with primary HPV, midwife-based liquid-based cytology sampling) and YE (current program with primary HPV, self-test option). All other types of tests, including opportunistic (non-organized) and/or symptom-prompted testing at e.g. a gynecologist office, are indexed with other prefixes by the sample taker, which allows for straight-forward classification of organized versus opportunistic testing. While the exact reasons for taking non-organised cervical tests are unknown, the non-organised samples are much more often positive for HPV and cytological abnormalities (compare the data on all samples to those of screening samples in Table 2).

Storage of samples follows the Swedish Biobank Law^20^. All screening participants receive written information about the sample storage and how to opt out of storing their samples for quality assurance and research. Participating in screening without actively opting out is the consent required by law.

Each region in Sweden has a regional biobank centre that can receive opt-out requests for all biobanks at any time. The cohort thus both collects information on opt-out at enrolment and receives requests for later retraction of consent. If a retraction of consent is received, the sample is destroyed, and a notification is sent to the woman’s address.

The law allows the use of biobanked samples for research purposes, provided that the Swedish Ethical Review Authority has vetted each research project. The Ethical Review Authority is appointed directly by Sweden’s government and chaired by a senior judge.

The typical cervical sample is immersed in 20 ml methanol-containing fixative, from which 0,005 to 4 ml may be used for clinical diagnostics (HPV tests and cytology analyses). The remaining 16 ml is concentrated to a 600-microliter storage volume and stored at −30 °C in a 96-well format. The concentration and labelling procedures are automatic, ensuring uniformity and minimising the risk of sample identity mix-ups. The identity of each sample is registered using a 2D barcode at the bottom of each storage vial, as well as the plate number and position of the vial. Intermittent quantification of human DNA using beta-globin real-time PCR is used for quality control. Extensive validation for metagenomics, epigenomics and genome-wide SNP genotyping is performed in the framework of research projects.

## Data Records

The Cervical Screening Cohort provides information about the primary results from testing cervical samples for HPV, cytology, and histopathology, and data on any secondary research analyses performed on the samples. The samples are available for external researchers, provided appropriate permissions from the Swedish Ethical Review Authority and the Region Stockholm Medical Biobank.

The following are the main components of the software platform used to support the screening program and manage the produced data:

- The Swedish National Cervical Screening Registry Analysis Database, NKCx (https://nkcx.se/index_en.htm) is based on Microsoft SQL Server. Data can be imported and exported as comma-separated values (CSV) files.
- In the Cytology Biobank, a Lab Information Management System (LIMS) manages the sample storage, importing and exporting data as CSV and Excel files.
- A Hopsworks software platform (https://www.hopsworks.ai/) is deployed at a computer cluster at Karolinska Institutet to perform metagenomics and machine learning analyses. Sequencing data and metadata are uploaded interactively or through Jupyter notebooks to facilitate metadata uploading and use. The published bioinformatics pipelines for metagenomics analyses, available through GitHub^17,18^, are also implemented in the Hopsworks platform at Karolinska Institutet.
- In-house developed JAVA-based management tools for self-testing, reporting and data extraction for research.

The cervical screening cohort’s data life cycle involves several relevant data components described in Table 1.

**Table 1:** Main data components of the cervical screening cohort’s data life cycle.

Data component	Description	Main data type
Person	The Person is a woman who receives an invitation. The national personal identification number (PIN) identifies the participant throughout the data cycle. Formats: YYYYMMDD-NNNN, YYYYMMDDNNNN, YYMMDD-NNNN, YYMMDDNNNN. Example: 19990505-1234, 199905051234, 990505-1234, 9905051234. The in-house management tools convert the PIN to any standard format.	Person ID: String
Invitation	According to the screening recommendations, regional units and the CCCE send invitations to the women. The invitations are recorded in the NKCx database. The invitation is a pdf file stored in a CCCE server, and it is sent as a digital letter or as a physical letter.	Invitation: Text, PDF Invitation date: Date Person ID: string
Consent	The consent is managed by the CCCE in-house system and linked to the participant. It is a record that includes text, date, PIN, what is consented to, and information about the project responsible.	Person ID: string Content: text Consent Date: Date Consent: Yes, No
Sampling	Sampling is the process of taking the sample from the participant. The in-house system registers sampling records containing PIN, date, sampling place, nurse (midwife) and notes. In recent years, self-sampling is increasingly used.	Person ID: string Date: Date Place: Text Sampling type: text Nurse: String
Sample	The sample is sent from the clinic (or by regular mail if it is a self-taken sample) to the CCCE lab, where it is stored and analysed. The main fields of the record are sample ID, sample type, participant’s PIN, storage location and condition.	Sample ID: string (QR, bar codes) Person ID: string Sample type: string Storage location: structure data (string)
Analysis	The analyses done on cervical samples are always HPV, in some cases cytology and histopathology, if it is a cervical biopsy. The record for an analysis contains the analysis ID, sample ID, participant’s PIN, and the analysis name.	Analysis ID: string Sample ID: string Analysis type: String Person ID: string
Analysis result	The analysis results are registered in the NKCx and the CCCE in-house database. The record contains the result ID, analysis ID, sample ID and Person ID. In the in-house system and NKCx, the results are registered as SNOMED codes.	Result ID: string Analysis ID: string Result: String Person ID: string

### Cervical screening cohort data

The Swedish Cervical Screening Cohort is updated yearly. Table 2 provides updated information to the end of 2021: Number of enrolled women, number of sampling occasions and the number of cytology and HPV tests and their results.

**Table 2:** The content of the Swedish Cervical Screening Cohort. The number of samples from different screening occasions is larger than the number of unique women, because women are screened again after a screening interval has passed. The tabel also displays the number of samples from organised screening and the tital number of samples (including samples from non-organised cervical sampling) and the results of the cytological analyses and the HPV testing.

All samples		
Ages	Number of women with samples	Number of samples (from different sampling dates)
23–30	214,575	317,020
31–49	359,743	632,641
50–70	191,906	279,008
All	673,257 (663,387 in above age groups)	1,243,006 (1,228,669 in above age groups)

Screening samples		
Ages	Number of women with samples	Number samples
23–30	200,605	268,607
31–49	335,761	539,920
50–70	172,744	232,371
All	624,348 (623,993 in above age groups)	1,040,936 (1,040,898 in above age groups)

All samples				
	Cytology results		HPV-results	
	Normal cytology	Abnormal cytology	Negative	Positive
Ages				
23–30	225,126	34,815	67,994	42,883
31–49	265,583	32,865	362,731	56,576
50–70	97,003	9,119	191,053	19,074
All	599,639 (587,712)*	78,833 (76,799)	627,902 (621,778)	120,294 (118,533)

Screening samples				
	Cytology results		HPV-results	
	Normal cytology	Abnormal cytology	Negative	Positive
Ages				
23–30	190,344	21,657	55,045	29,417
31–49	188,454	18,346	327,612	42,814
50–70	56,562	3,746	168,789	13,402
All	435,366 (435,360)	43,751 (43,749)	551,476 (551,446)	85,637 (85,633)

*Numbers in () are the sum of the specified age groups.

The dataset comprising all the samples from 2011 to 2021 (Table 2) is openly available from the *figshare* repository, and includes the variables presented in Table 3^21^.

- 10.6084/m9.figshare.24799524.v1
- Link: 10.6084/m9.figshare.24799524.v1

**Table 3:** Variables in the available dataset in *figshare*.

Variable	Description of variables
Sequence_number_women	External identifier to link a woman with her samples
Sequence_number_sample	External identifier to link samples to a woman
Sample_year	Sampling year
Age	Age range (8 years)
Screening_sample	“Yes”: Organized screening, “No”: Another cell test
Cytology_test	“1” if the test was done
HPV_test	“1” if the test was done
Cytology_result	“Normal” or “Abnormal” if Cytology_test is “1”
HPV_result	“Negative” or “Positive” if HPV_test is “1”

Samples in the *figshare* dataset^21^ can be on-demand linked to the NKCx registry data to get insight about the donors. The related variables in the NKCx registry are presented in Supplementary Table 1.

## Technical Validation

The NKCx is annually linked to the Swedish Population Registry to compare the Personal Identification Numbers and ensure that only data from existing residents and living women are registered. It is both a measure of accuracy and completeness, as the number of women who are resident in Sweden but have no data in the screening registry is small (<3% of the total population). Other data, such as the high cervical cancer incidence among never-attenders, suggest that the few per cent of women with no data in the registry is not a case of missing data but probably reflects true non-participation.

The samples in the cervical cohort are used yearly for a so-called audit of cases of cervical cancer and high-grade precursors. Suppose a woman is diagnosed with cervical cancer or a high-grade precursor. In that case, the registry is queried for prior samples from these women. These samples are then retrieved from the cohort sample collection and subjected to extended testing to investigate possible reasons for false negative screening analyses^12^. It does happen that a sample cannot be localised in the storage, but this is rare (none of the 488 samples in reference 11). A sample may also have unsuitable quality for new PCR, but this is rare (1 out of 476 samples in reference 11).

## Usage Notes

A complete description of the NKCx variables is registered in the Swedish Research Council metadata catalogue (www.registerforskning.se). It is accessible by requesting an account. An example of the NKCx database variables is provided in supplementary Table 1.

Some aggregated data (not identifiable) are openly accessible through https://nkcx.se/qind_e.htm. It is possible to request aggregated data from the registry chairperson (https://nkcx.se/contact_e.htm). There are no restrictions for requesting aggregated data for research and other purposes. Indeed, requests for aggregated data by the mass media are relatively frequent.

Access to identifiable, individual-level data is also possible for research. It requires:

- Permission from the Swedish Ethical Review Authority. Applying to the Swedish Ethical Review Authority requires at least one Swedish citizen to be a co-applicant. International teams are encouraged to contact the registry at info@nkcx.se, and the registry will then assist with preparing the application.
- Permission from the Steering Group of the NKCx. The application forms and instructions are at https://nkcx.se/research_en.htm.

Access to pseudo-anonymised and aggregated sample data is possible through *figshare* (10.6084/m9.figshare.24799524.v1)^21^.

The procedure to obtain data associated with the samples is the same as for accessing identifiable data (as explained above). In addition, it requires permission from the regional biobank centre of Region Stockholm. The process for such permissions is somewhat complicated. Usually, the NKCx staff help the requesting scientist to prepare and file the biobank retrieval application.

If complementary data from other registries are needed, NKCx can offer researchers advice on obtaining such data through registry linkages.

### Supplementary information

Supplementary Table 1

## Data Availability

The cervical screening data workflow uses different software components and assigns custom identifiers along the process. However, all data associated with a participant (invitation, sampling, analysis) are linked through the participant’s national personal identification number. This code can be accessed only by researchers and stakeholders authorised by the Swedish Ethical Review Authority. The data can be pseudo-anonymised and aggregated before sharing, depending on the intended use.
