# Supplementary material for: The Swedish Cervical Screening Cohort
Source: Sci Data. 2024 Jun 26;11:697. doi: 10.1038/s41597-024-03519-2 (PMC11208431; doi:10.1038/s41597-024-03519-2)
Supplement: Supplementary file 1 — Supplementary Table 1 [file 41597_2024_3519_MOESM1_ESM.docx]

Supplementary Table 1

| **Variable** | **Description of variables** |
| --- | --- |
| **Table: NKC_CELL_6922** | **Cytology data from 1969 until 2022** |
| AGE | Age |
| ANS_CLINIC | The clinic responsible for diagnostic report |
| COUNTY_ID | County's id, the county which the tests/histology were taken in |
| DEB_CLINIC | The identification for the laboratory |
| DIAG_ID | The identification of diagnosis report |
| LABORATORY_ID | The identification for the laboratory |
| DIFF_DAYS | The days between sample date and response date |
| PERSON_ID | Person-id (pseudonymised used instead of PNR) |
| REFERRAL_NR | Referral number |
| REF_SITE | Clinic |
| REG_DATE | Date of registration |
| REFERRAL_TYPE | Type of Referral |
| REM_CLINIC | The clinic which sends referral to for the patient |
| RESIDC | County |
| RESIDK | Municipal code |
| RESPONSE_DATE | Response date for sample |
| SAMPLE_DATE | Date of sample |
| SAMPLE_ID | The identification number of sample |
| SAMPLE_YR | Year of sample |
| SCR_TYPE | Type of screening 1. Organised screening 2. Another test |
| SNOMED | SNOMED codes for diagnosis (SNOMED) |
| X_REG_DATE | New variable for date of registration (The cleaned variable which is derived from REG_DATE) |
| X_RESPONSE_DATE | New variable for response date (cleaned data) in date format |
| X_SAMPLE_DATE | New variable for sample date (the cleaned variable, is derived from SAMPLE_DATE) |
| X_SAMPLE_YR | New variable for the year of sample (The cleaned variable which is derived from SAMPLE_YR) |
| X_SNOMED | New variable for SNOMED code (The cleaned variable which is derived from SNOMED) |
| **Table: NKC_EXT_HPV** | **HPV data from different microbiological labs** |
| AGE | Age |
| CLINIC | Clinic |
| COUNTY_ID | County's id |
| DATASRC | Screening labs for Microbiology HPV tests ((MA: Malmö, ALE: Aleris, STH: Stockholm, SMA: Småland, GBG: Göteborg)) |
| HPV_TYPE | The type of HPV (16, 32, …) |
| HPVDIAG | HPV status |
| LAB_ID | Lab-id |
| LABORATORY_ID | The identification for the laboratory |
| PERSON_ID | Person-id |
| REFERRAL_NR | Referral number |
| REFERRAL_NR2 | External referral number |
| REFERRAL_TYPE | Type of Referral |
| RESPONSE_DATE | Response date for HPV test |
| SCR_TYPE | Type of screening 1. Organised screening 2. Another test |
| TRANSLATION | The type of HPV and translation of HPV as result |
| X_REG_DATE | The date of registration (cleaned data for reg_date) |
| X_SAMPLE_DATE | The date of sample |
| X_SAMPLE_YR | The year of sample |
| **Table: NKC_HPV** | **HPV data from routine clinical labs** |
| AGE | Age |
| ANS_CLINIC | The clinic responsible for diagnostic report |
| COUNTY_ID | County's id |
| DEB_CLINIC | The clinic which charges the sample reimbursement |
| DIAG_ID | Diagnostic report id |
| HPVDIAG | HPV status |
| LABORATORY_ID | The identification for the laboratory |
| PERSON_ID | Person-id |
| REF_SITE | Clinic |
| REFERRAL_NR | Referral number |
| REFERRAL_TYPE | Type of Referral |
| REG_DATE | Register date |
| REM_CLINIC | The clinic which sends referral for the patient |
| RESIDC | County |
| RESIDK | Municipal code |
| RESPONSE_DATE | Response date for HPV test |
| SAMPLE_DATE | Sample's date |
| SAMPLE_ID | Sample's id |
| SAMPLE_TYPE | Type of sample |
| SAMPLE_YR | Sample's year |
| SCR_TYPE | Type of screening 1. Organised screening 2. Another cell test |
| SNOMED | SNOMED code (diagnose code) |
| TRANSLATION | The type of HPV and translation of HPV as result |
| X_REFERRAL_TYPE | The new variable for referral_type |
| X_REG_DATE | The date of registration (cleaned data for reg_date) |
| X_RESPONSE_DATE | The new variable for response date (cleaned variable) |
| X_SAMPLE_DATE | The date of sample (cleaned variable) |
| X_SAMPLE_YR | The year of sample |
| X_SNOMED | The cleaned variable which is used instead of SNOMED |
| **Table: NKC_INV_9322** | **Invitations from year 1993 until 2022** |
| AGE | Age |
| CANCEL_DATE | The date of cancelling of invitation |
| DEREG_REASON | The reason for deregistering of invitation |
| DISTRICT | Part of a larger geographical area |
| INVITATION_ID | Invitation's id |
| INV_DATE | The date of invitation |
| INV_TYPE | Type of invitation |
| INV_YEAR | The year of invitation |
| LABORATORY_ID | The identification for the laboratory |
| PERSON_ID | Woman's id |
| RCC | Indicator to know if RCC sends invitation data 1=Yes; .=No |
| RESIDC | County |
| RESIDF | Parish |
| RESIDK | Municipal code |
| SELFTEST | Self sample=1 is for HPV-self sample invitation |
| X_INV_DATE | The new variable for date of invitation (The cleaned data which is derived from INV_DATE) |
| X_INV_YR | The new variable for year of invitation (The cleaned data which is derived from INV_YR) |
| **Table: NKC_INV_KIT_1921** | **Invitations to self-test (HPV-Kit) from year 2019 until 2022** |
| AGE | Age |
| CANCEL_DATE | The date of cancelling of invitation |
| DEREG_REASON | The reason for cancel of invitation to self-test (HPV-Kit) |
| DIRECT_SEND | If invitation is related to direct sent HPV-KIT or not |
| DISTRICT | Part of a larger geographical area |
| INV_DATE | The date of invitation |
| INV_TYPE | Type of invitation |
| INV_YEAR | The year of invitation |
| INVITATION_ID | Invitation's id |
| LABORATORY_ID | The identification for the laboratory |
| PERSON_ID | Woman's id |
| RCC | Indicator to know if RCC sends invitation data 1=Yes; .=No |
| RESIDC | County |
| RESIDK | Parish |
| X_INV_DATE | Municipal code |
| X_INV_TYPE | The type of invitation (when there are other types in data, we classify the invitation as Cancelled/Blocked, Invitation, Reminder |
| X_INV_YR | The new variable for year of invitation (The cleaned data which is derived from INV_YR) |
| **Table: NKC_PAD_6922** | **Histopathology test data** |
| AGE | Age |
| ANS_CLINIC | The clinic which sends response |
| COUNTY_ID | County's id, the county which the smears/histology were in |
| DEB_CLINIC | The clinic which charges the samples |
| DIAG_ID | The id of diagnose |
| DIAG_NR | Diagnosis code's number |
| DOCTOR | Doctor |
| LABORATORY_ID | The identification for the laboratory |
| OBLITERATED | Cancelled test for pad (Y=Yes; or ‘N'=No) |
| PERSON_ID , PNR | Person-id (used instead of Person nr.) |
| REFERRAL_NR | Referral number |
| REFERRAL_TYPE | Type of Referral |
| REF_SITE | Clinic |
| REG_DATE | Date of registration |
| REM_CLINIC | The clinic which sends referral for patient |
| RESIDC | County |
| RESIDK | Municipal code |
| RESPONSE_DATE | Response date |
| SAMPLE_DATE | The date of sample |
| SAMPLE_ID | The identification of sample |
| SAMPLE_NR | Sample number |
| SAMPLE_TYPE | Type of sample 1. Organised screening 2. Another cell test |
| SAMPLE_YR | The year of sample |
| SNOMED | Diagnose code (SNOMED) |
| TOPO | Topology of the biopsy code |
| X_REG_DATE | New variable for date of registration (The cleaned variable which is derived from REG_DATE) |
| X_RESPONSE_DATE | The new variable for response date (cleaned variable) |
| X_SAMPLE_DATE | New variable is used instead of sample_date (The cleaned variable which is derived from SAMPLE_DATE) |
| X_SAMPLE_YR | New variable is used instead of SAMPLE_YR (The cleaned variable which is derived from SAMPLE_YR) |
| X_SNOMED | New variable for SNOMED code (The cleaned variable which is derived from SNOMED) |
| **Table: NKC_PERSON** | **Women's birth date and person-id** |
| BIRTH_DATE | Birth date (This variable is missing before year 1880) |
| BIRTH_YR | Birth year (This variable is missing before year 1880) |
| CREATE_DATE | The date of creation of new person-id |
| PERSON_ID | Person-id |
| VALID_PNR | Indicator for validate of person number |
| **Table: NKC_PAD_TRANSLATED** | **Translation of histopathology data** |
| AGE | Age |
| ANS_CLINIC | The clinic which sends response |
| COUNTY_ID | County's id |
| LAB_ID | Lab-id |
| PAD_CLASS | Classification of lesion |
| PAD_SEV | The severity of lesion |
| PERSON_ID | Person-id |
| REFERRAL_NR | Referral nr. |
| REFERRAL_TYPE | Referral type |
| REM_CLINIC | The clinic which charges the samples |
| RESIDK | Municipal code |
| RESPONSE_DATE | The date of response for sample |
| SAMPLE_ID | The identification of sample |
| SAMPLE_TYPE | The type of sample |
| SNOMED_ORIGINAL | The original SNOMED code |
| SNOMED_TRANSLATED | Standardised SNOMED code |
| TRANSLATED | Indicator 1. Translated SNOMED code 0. Not possible to translate the SNOMED code |
| TOPO3 | Topology of the biopsy (the first 3 characters) |
| X_REG_DATE | The date of registration should be used instead of reg_date |
| X_SAMPLE_DATE | The cleaned data should be used instead of sample_date |
| X_SAMPLE_YR | The year of sample |
| **Table: NKC_TRANS_CELL** | **Translated cytology sample data to standardised diagnostic code** |
| AGE | Age at diagnose |
| COUNTY_ID | County's id, the county which the smears/histologies were in |
| LAB_ID | Lab's id |
| LCODES | The combination of original codes |
| M00110 | If value M0010 is present in UCODES then column M00110 is set to 1 else 0 |
| M09005 | If value M09005 is present in UCODES then column M09005 is set to 1 else 0 |
| M09010 | If value M09010 is present in UCODES then column M09010 is set to 1 else 0 |
| M09019 | If value M09019 is present in UCODES then column M09019 is set to 1 else 0 |
| M69700 | If value M69700 is present in UCODES then column M69700 is set to 1 else 0 |
| M69710 | If value M69710 is present in UCODES then column M69710 is set to 1 else 0 |
| M69719 | If value M69719 is present in UCODES then column M69719 is set to 1 else 0 |
| M69720 | If value M69720 is present in UCODES then column M69720 is set to 1 else 0 |
| M74006 | If value M74006 is present in UCODES then column M74006 is set to 1 else 0 |
| M74007 | If value M74007 is present in UCODES then column M74007 is set to 1 else 0 |
| M76700 | If value M76700 is present in UCODES then column M76700 is set to 1 else 0 |
| M80009 | If value M80009 is present in UCODES then column M80009 is set to 1 else 0 |
| M80702 | If value M80702 is present in UCODES then column M80702 is set to 1 else 0 |
| M80703 | If value M80703 is present in UCODES then column M80703 is set to 1 else 0 |
| M80770 | If value M80770 is present in UCODES then column M80770 is set to 1 else 0 |
| M80772 | If value M80772 is present in UCODES then column M80772 is set to 1 else 0 |
| M81401 | If value M81401 is present in UCODES then column M81401 is set to 1 else 0 |
| M81403 | If value M81403 is present in UCODES then column M81403 is set to 1 else 0 |
| PERSON_ID | Person-id is used instead of PNR. |
| RESIDK | Municipal code |
| SAMPLE_ID | Sample id |
| SCR_TYPE | Type of screening 1. Organised screening 2. Another cell test |
| SNOMED_SEVERITY | The severity of the worst SNOMED code in Sample |
| SNOMED_WORST | The SNOMED code for the worst severity |
| UCODES | Unified codes. New column derived from column with same name in view TRANSLATION_MATRIX |
| X_REG_DATE | New variable for date of registration (The cleaned variable which is derived from REG_DATE) |
| X_SAMPLE_DATE | New variable for SAMPLE_DATE (The cleaned variable which is derived from SAMPLE_DATE) |
| X_SAMPLE_YR | Sample year (derived variable from SAMPLE_YR) |
| **Table: NKC_TRANS_CELL_DIAG** | **Translation table for pap smears per diagnose** |
| AGE | Age |
| ANS_CLINIC | The clinic sends response |
| BIRTH_DATE | Birth date |
| COUNTY_ID | County's id |
| LAB_ID | Lab-id |
| LABORATORY_ID | The identification for the laboratory |
| PERSON_ID | Person-id |
| REFERRAL_NR | Referral number |
| REFERRAL_TYPE | Type of Referral |
| REM_CLINIC | The clinic which sends referral for the patient |
| RESIDK | County |
| SAMPLE_ID | Sample's id |
| SCR_TYPE | Type of screening 1. Organised screening 2. Another cell test |
| TRAN_ID | The translation-id for SNOMED code |
| TRAN_RULE | The rule /source for translation of SNOMED ('lab'=if the lab uses the translation for SNOMED code, 'NA' if the SNOMED code is not translated |
| TRAN_SNOMED | The translation of SNOMED code |
| X_REG_DATE | The date of registration (cleaned data for reg_date) |
| X_SAMPLE_DATE | The date of sample |
| X_SAMPLE_YR | The year of sample |
| X_SNOMED | The cleaned variable which is used instead of SNOMED |

NKCx variable descriptions.
